# Multi-Omic Dynamics Associate Oxygenic Photosynthesis with Nitrogenase-Mediated H_2_ Production in *Cyanothece* sp. ATCC 51142

**DOI:** 10.1038/srep16004

**Published:** 2015-11-03

**Authors:** Hans C. Bernstein, Moiz A. Charania, Ryan S. McClure, Natalie C. Sadler, Matthew R. Melnicki, Eric A. Hill, Lye Meng Markillie, Carrie D. Nicora, Aaron T. Wright, Margaret F. Romine, Alexander S. Beliaev

**Affiliations:** 1 Chemical and Biological Signature Science, Pacific Northwest National Laboratory, Richland, WA 99352; 2 Biological Sciences Division, Pacific Northwest National Laboratory, Richland WA, 99352.

## Abstract

To date, the proposed mechanisms of nitrogenase-driven photosynthetic H_2_ production by the diazotrophic unicellular cyanobacterium *Cyanothece sp*. ATCC 51142 have assumed that reductant and ATP requirements are derived solely from glycogen oxidation and cyclic-electron flow around photosystem I. Through genome-scale transcript and protein profiling, this study presents and tests a new hypothesis on the metabolic relationship between oxygenic photosynthesis and nitrogenase-mediated H_2_ production in *Cyanothece* 51142. Our results show that net-positive rates of oxygenic photosynthesis and increased expression of photosystem II reaction centers correspond and are synchronized with nitrogenase expression and H_2_ production. These findings provide a new and more complete view on the metabolic processes contributing to the energy budget of photosynthetic H_2_ production and highlight the role of concurrent photocatalytic H_2_O oxidation as a participating process.

Photobiological H_2_ production is still a nascent technology with long-term potential for sustainable energy production with a low environmental impact. Although direct biophotolytic H_2_ production has been documented and studied for decades^1^, significant challenges remain for the development of microbial strains and conditions that can directly and efficiently use sunlight and water to produce H_2_. Chief among them is the low production rate, which is largely due to feedback inhibition of the H_2_ producing enzymes by O_2,_ an obligate byproduct of oxygenic photosynthesis. Limitations imposed by O_2_ sensitivity of the native hydrogenase and nitrogenase enzymes have motivated significant efforts to identify and even engineer O_2_ tolerant variants^2^ and multi-stage processes that temporally separate O_2_ and H_2_ evolution^3^. However, to date, the kinetic rates and sustainability of hydrogenase-mediated H_2_ production are low in comparison to those reported for some diazotrophic organisms that produce H_2_ in oxic-environments as a byproduct of nitrogenase catalyzed N_2_ fixation^4^,^5^,^6^.

Nitrogen-fixing cyanobacteria have been recognized as one of the most promising photolytic platforms for sustainable H_2_ production^7^,^8^,^9^,^10^,^11^. A unicellular marine strain *Cyanothece sp*. ATCC 51142 (hereafter *Cyanothece* 51142) has emerged as a model system because of its ability to produce H_2_ at rates > 100 μmol-H_2_ hr^−1^ mg-Chl^−1^ under photosynthetic conditions associated with continuous illumination^7^,^12^. Although recent genome-enabled studies have provided systems-level insights into the mechanisms governing diurnal growth and metabolism of *Cyanothece* 51142^7^,^13^,^14^,^15^,^16^,^17^, the processes that support H_2_ production in this organism have yet to be fully resolved. The current and prevailing view assumes that H_2_ production mediated by energetically expensive nitrogenase activity in *Cyanothece* 51142, and other closely related strains, is exclusively supported by ATP and reductant derived from oxidation of intracellular glycogen and/or cyclic-electron flow around photosystem (PS) I^7^,^13^,^18^. Here we present evidence to support a new model whereby energy derived directly from oxygenic photosynthesis (i.e., linear electron flow through PS II) is an important process in funding the energy budget required for nitrogenase activity under illuminated, nitrogen-deplete conditions. These conclusions are supported by a combined analysis of detailed process and integrated transcriptome and proteome profiles across a photosynthetically driven H_2_ production process.

## Results

### H_2_ production kinetics

**Figure 1:**
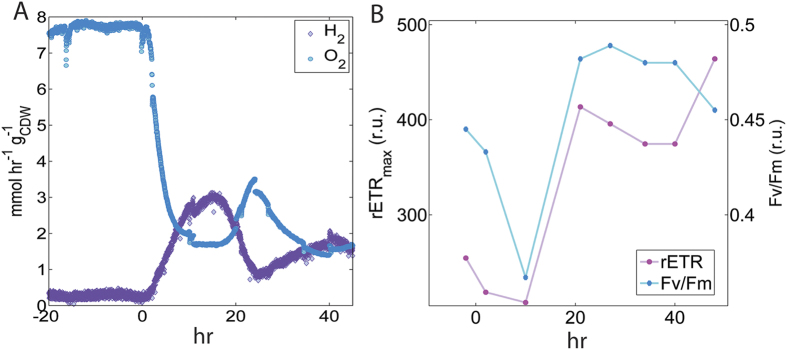
(**A**) Specific rates of net H_2_ and O_2_ production. Time zero indicates onset of nitrogen-depletion and absence of media addition (dilution rate = 0). (**B**) Variable chlorophyll fluorescence originating from PS II: the maximum relative electron transfer rate (rETR_max_) and the optimal quantum yield (Fv/Fm). Data plotted at times prior to t = 0 represent measurements taken during the ammonia limited chemostat (i.e., steady-state precondition).

High levels of H_2_ production were achieved by interrupting medium flow to an ammonium-limited chemostat of *Cyanothece* 51142, grown in an Ar/CO_2_ atmosphere under continuous illumination (Fig. 1). The resulting N-depletion yielded metabolically active but non-growing cells, as confirmed by constant cell dry weight (CDW) concentrations (0.086   0.005 g_CDW_ L^−1^) (Fig. S1). The maximum specific rate of H_2_ production (q_H2_) by *Cyanothece* 51142 was reached after 14.5 hours of N-depletion and measured to be 3.12 mmol-H_2_ hr^−1^ g^−1^_CDW_ (279 μmol-H_2_ mg-Chl *a*^−1^ hr^−1^) which, to our knowledge, is the highest reported rate of H_2_ production per unit biomass under photoautotrophic conditions^7^. H_2_ production persisted throughout the experiment and is known to be sustainable through this method for more than 100 hours^12^. The average specific rates were 2.24 and 1.70 mmol-H_2_ hr^−1^ g^−1^_CDW_ over 20 and 40 hours, respectively. Notably, the *Cyanothece* 51142 cells were maintained under continuous irradiance under all culturing steps; hence, H_2_ production was not dependent on diel cycling as reported in previous studies^7^,^14^.

### Concurrent oxygenic photosynthesis

In this study, H_2_ production was only measured under aerobic conditions maintained by active oxygenic photosynthesis (i.e., PS II activity) in the bioreactor environment. Dissolved O_2_ concentrations ranged from steady-state values of 10 μM to 2.4 μM, observed during H_2_ production. As this specific rate of O_2_ production (q_O2_) is non-zero and represents the net rate of photosynthesis, whereby the gross rate of O_2_ evolution is greater than the rate of respiration^19^; concurrent observations of O_2_ and H_2_ production provide direct evidence of energy acquisition from photocatalytic H_2_O oxidation via linear electron flow through PS II. Electrons originating from H_2_O are available for photosynthetic electron transport, which can result in proton reduction and generation of the proton motive force. The q_O2_ values ranged from 7.5 ± 0.2 mmol-O_2_ hr^−1^ g^−1^_CDW_ to 1.4 mmol-O_2_ hr^−1^ g^−1^_CDW_ for steady-state and N-deplete conditions, respectively (Fig. 1). Net photosynthesis rates decreased immediately upon initiation of N-depletion but then increased inversely with q_H2_. The capacity for linear electron flow through PS II was also confirmed via measurements of variable Chl *a* fluorescence (Fig. 1B). The maximum relative rates for electron transport (rETR_max_) increased throughout the H_2_ production profile and at their lowest levels were near the values measured from the steady-state precondition. The optimal photochemical quantum yield of PS II (Fv/Fm) decreased initially with the onset of N-depletion but then increased beyond the steady-state value for the remainder of the H_2_ production period. Evidence for cyclic electron flow, as inferred from rises in post-illumination Chl *a* fluorescence (Fig. S2) was only observed during the ammonium-limited chemostat precondition.

### Global transcriptional and translational dynamics

**Figure 2:**
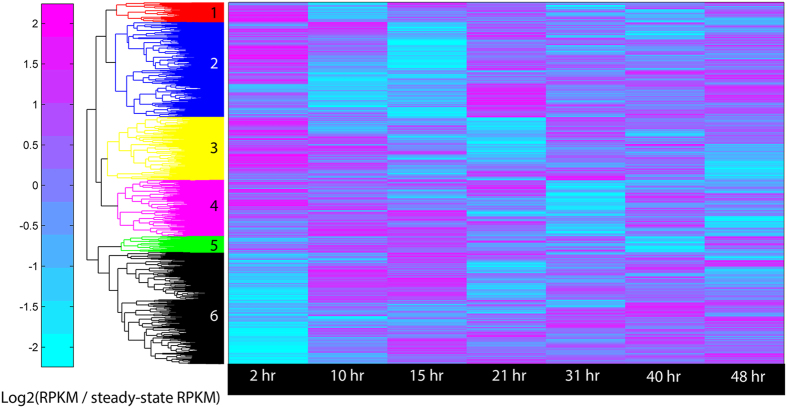
Hierarchical clustering of the relative mRNA abundances given in reads per kilobase per million reads (RPKM) normalized by steady-state. 3319 unfiltered genes (of 5303) categorized into six main cluster groups: 1-red (181 genes), 2-blue (872 genes; enriched for PS I and electron transport, p < 0.05), 3-yellow (580 genes), 4-magenta (516 genes), 5-green (146 genes) and 6-black (1024 genes; enriched for PS II and N-fixation, p < 0.05).

Absolute and relative expression of key metabolic processes varied temporally across eight time-resolved sample points within the H_2_ production profile. Dynamic expression patterns of energy (catabolic and photosynthetic) and nitrogen metabolism genes were observed within the global transcriptome (Fig. 2). The transcriptome was directly compared to and showed general agreement with the global proteome patterns (Fig. S3), which was not entirely unexpected in pre-synchronized, non-growing cells. Enrichment of key functional roles identified correlations between photosynthesis and nitrogen fixation processes (Table S1 and S2): transcripts specific for the main subunits for PS I were enriched (p < 0.004) in a separate group (Fig. 2; blue) and distinct from the main subunits of PS II, which co-clustered (p < 0.05) with the nitrogen fixation machinery (Fig. 2; black).

### Nitrogenase and hydrogenase expression

**Figure 3:**
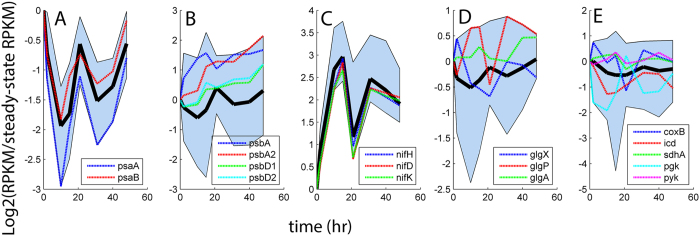
Relative abundance profiles of functionally categorized gene transcripts. Solid black lines indicate the mean profile taken from each collection of genes. Edges of each shaded region represent the respective maximum and minimum relative abundances of transcripts measured at each sampling point. (**A**) PS I (11 *psa* genes); (**B**) PS II (31 *psb* genes); (**C**) Nitrogenase (20 *nif* genes); (**D**) Glycogen metabolism (8 *glg* genes); (**E**) Catabolic energy metabolism (18 genes involved in glycolysis, TCA and oxidative phosphorylation).

**Figure 4:**
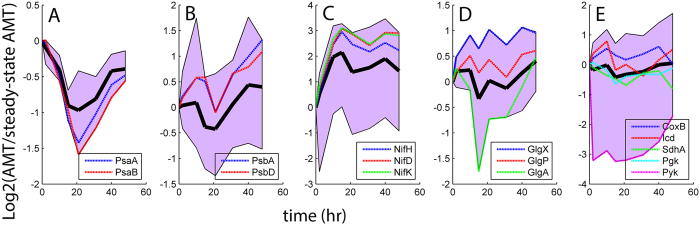
Relative abundance profiles of functionally categorized proteins. Solid black lines indicate the mean profile taken from each collection of proteins. Edges of each shaded region represent the respective maximum and minimum relative abundances of proteins measured at each sampling point. (**A**) PS I (6 Psa proteins); (**B**) PS II (15 Psb proteins); (**C**) Nitrogenase (12 Nif proteins); (**D**) Glycogen metabolism (7 Glg proteins); (**E**) Catabolic energy metabolism (13 proteins involved in glycolysis, TCA and oxidative phosphorylation).

Transcriptional and translational expression patterns of *nif* genes, as compared to those displayed by the bi-directional hydrogenase (Hox), implicated nitrogenase as the key catalytic process for H_2_ production in this study. This result is consistent with other studies reporting the physiology of *Cyanothece* 51142 functioning within N-limited (or deplete) environments^7^,^20^. The relative transcript and protein abundance profiles containing all of the *nif* transcripts, were positively correlated (R^2 ^> 0.71) with the multi-modal (damped oscillatory) pattern of H_2_ production (Fig. 3C and S4). In general, Nif protein expression corresponded with the transcriptional dynamics and was significantly increased during H_2_ production (Fig. 4C). The abundances (RPKM) for the encoding transcripts and NifHDK proteins were very high (compared within the global expressome) during chemostat growth as well as throughout H_2_ production (see supplementary data file). At the same time, the relative abundances of transcripts for the uptake hydrogenase (*hup*LS) varied during H_2_ production (Fig. S5), displaying no distinctive patterning with respect to H_2_ or O_2_ production. The bidirectional hydrogenase (*hox*) genes exhibited significantly lower RPKM values than *nif* (e.g., *hoxH* was only 0.15% of *nifH* at max q_H2_) and the relative abundances did not cluster uniformly or share common profile patterning with H_2_ production or photosynthesis. Unique peptides were not detected for either the Hup or Hox proteins; hence they were not represented within the global proteome.

### Photosynthetic units

The relative abundances of the PS II reaction center transcripts and proteins (PsbA and PsbD) increased with H_2_ production (Figs 3B and 4B), while relative abundances of PS I reaction centers (PsaA and PsaB) were generally decreased (Figs 3A and 4A). Accordingly, the relative transcript abundances of genes encoding PS II and PS I components displayed positive and negative correlation (R^2 ^> 0.6) with nitrogenase expression, respectively (Fig. S6). Transcript and protein levels of PS II genes correlated with each other and increased with H_2_ production. In contrast, relative abundance profiles for all PS I transcripts showed multi-modal (oscillatory) patterning (Fig. 3A). The dynamics of PS I proteins differed from the transcripts and displayed mono-modal behavior which was inversely correlated with the net O_2_ production at ~21 hr (Figs 1 and 4A). Taken together with the net photosynthesis rates and Chl *a* fluorescence kinetics (Fig. S2), expression dynamics of PS II and PS I machinery provides key evidence linking the H_2_ production in *Cyanothece* 51142 with increase in linear electron flow through and concurs with decreased capacity for cyclic electron flow as the dominant ATP generating process.

### Energy and glycogen metabolism

The dynamic patterns of key glycogen metabolism pathways were variable and did not correlate with the nitrogenase expression at either the transcriptional or translational levels (Fig. 3D and S6). Evidence for glycogen degradation was observed in the expression profiles of the glycogen debranching enzyme (*glgX*; cce_3465) and glycogen phosphorylase (*glgP*, cce_1629), which showed increased abundances relative to the ammonium limited precondition and positive correlation with nitrogenase expression at the translational level (Fig. 4D and S6). However, relative abundance patterns of these glycogen degradation genes were decreased at the transcriptional level. Glycogen syntheses genes revealed opposite expression dynamics as compared to degradation and the relative protein abundance of glycogen synthase (GlgA; cce_3396) was significantly decreased during peak H_2_ production (Fig. 4D).

The principal processes involved in catabolic energy metabolism, downstream of glycogen degradation, generally showed decreased relative abundances during H_2_ production and negative correlation with nitrogenase expression (Fig. S6). The relative protein abundance profiles of the two ATP generating steps of glycolysis (phosphoglycerate kinase and pyruvate kinase; cce_4219 and cce_3420, respectively) were significantly decreased (p < 0.05; Fig. 4E). Expression at the transcriptional level (*pgk* and *pyk*) was more varied but generally demonstrated a decrease across the H_2_ production profile (Figs 3E and 4E). The relative transcript and protein abundance profiles for the key reductant generating reactions of the tricarboxylic acid cycle (TCA), such as isocitrate dehydrogenase (Icd; cce_3202) and succinate dehydrogenase (SdhA; cce_0663), were generally decreased (Figs 3E and 4E); although some variability was observed in the Icd protein profile during the early time points of the H_2_ production profile. A principal gene in respiration and oxidative phosphorylation for *Cyanothece* 51142 is cytochrome c oxidase (*coxB*; cce_1977). The relative abundance profiles of CoxB proteins generally increased with H_2_ production (Fig. 4E) but showed very weak correlation with nitrogenase expression (Fig. S6). The transcriptional profiles for *coxB* did not correlate with nitrogenase expression and showed relative decreases during the local net-photosynthesis minima corresponding to peak H_2_ production (Figs 1 and 3E).

## Discussion

The results of this study yield kinetic and genome-scale evidence that supports a new level of insight into the metabolic processes that supply the energy requirements for nitrogenase-mediated H_2_ production in *Cyanothece* 51142. These results frame the concept of active reductant and ATP generation originating from a combination of energy metabolism processes including linear electron flow through PS II. The H_2_ production profiles measured under these experimental conditions correlate positively with nitrogenase and PS II expression but negatively with these of PS I and some of the key catabolic processes required to harvest energy from intercellular glycogen stores. In addition, expression of the principal downstream ATP and reductant generating steps of glycolysis and the TCA cycle were generally decreased relative to the nitrogen limited steady-state precondition. Taken together, these results suggest that the high levels of ATP and reductant required to continuously support nitrogenase catalyzed H_2_ production do not originate solely from glycogen catabolism but also from linear electron flow through PS II. This is an exciting development, since it has been previously reported and generally assumed that H_2_ production, via nitrogenase activity in *Cyanothece* 51142 and highly related strains, is exclusively supported by ATP and reductant derived from glycogen degradation and/or cyclic-electron flow^7^,^13^,^18^,^21^.

The importance of linear electron flow through PS II, as it relates to supporting nitrogenase driven H_2_ production in *Cyanothece* 51142, has been a topic of scientific ambiguity. Multiple studies have investigated the effect of PS II inhibition with 3-(3,4-dichlorphenl)-1,1-dimethylurea (DCMU) during N-depleted H_2_ production. It has been reported that the addition of DCMU to Ar-flushed *Cyanothece* 51142 suspensions had no effect on H_2_ production^7^. However, another study (that supports the current hypothesis) reported that the addition of DCMU to cells had delayed but dramatic inhibitory effect of H_2_ production and a similar effect was observed after addition of the electron transport inhibitor p-benzoquinone (BQ), a plastoquinone analog^12^. The inference from these studies was that cyclic electron flow around PS I was the key mechanism supplying ATP for nitrogenase activity. However, the comprehensive gene and protein profiles presented here show that expression of critical subunits for energy acquisition in PS I (PsaA and PsaB) are decreased compared to the reaction centers of PS II (PsbA and PsbD). Additionally, peak PS II performance, as inferred from variable Chl *a* fluorecence parameters rETR_max_ and Fv/Fm, was observed when the relative abundance PS I reaction center proteins were lowest (~20 hr). Hence, reduced expression of PS I did not restrict linear electron flow. This strongly suggests the involvement of PS II in nitrogenase-mediated H_2_ production. In contrast to prior belief, it is likely that ATP generation is primarily facilitated by linear electron flow through each photosystem accounting for the full potential for proton translocation, ATPase activity and generation of reducing equivalents.

Although changes in expression patterns do not directly represent enzymatic activity, the H_2_ production and photosynthesis kinetics are in strong agreement with the global transcript and protein measurements. The current results are in agreement with previously reported Chl *a* fluorescence kinetics, across similar H_2_ production profiles, which also revealed that the maximum electron transfer rates (ETR_max_) increased substantially along the H_2_ production profiles under different carbon availability regimes^12^. This evidence complements the *in situ* reaction measurements that show the gross rate of oxygenic photosynthesis exceeding the rate of respiration which confirms that linear electron flow through PS II is active during nitrogenase mediated H_2_ production. This result is in contrast to observations made in cyanobacterium *Trichodesmium* which has been observed to facilitate nitrogen fixation concurrently with oxygenic photosynthesis by scavenging O_2_ via the PS I mediated Mehler reaction, resulting in negative net-O_2_ production rates^22^. We note that results from the current study only represent the physiology and metabolic potential of *Cyanothece* 51142 under a specific photobioreactor-facilitated H_2_ production process, which is not directly comparable to any natural cyanobacterial ecosystem.

Nitrogenase-mediated H_2_ production under aerobic conditions has been observed for *Cyanothece* 51142 in a number of reported studies^7^,^12^. However, previous reports assumed that glycogen acts as the electron donor for oxidative phosphorylation and cyclic-electron flow^7^. The expression patterns for enzymes required for catabolic energy acquisition within glycolysis (phosphoglycerate kinase and pyruvate kinase) and the TCA cycle (isocitrate dehydrogenase and succinate dehydrogenase) do not support this previously proposed scheme. It is unlikely that glycogen serves as the sole electron donor to nitrogenase while there is a net-positive oxygenic photosynthesis rate; as observed under the conditions reported in this study. Supporting evidence for this realization has previously been presented in a similar study reporting that intracellular carbohydrates levels (glucose equivalents) and glycogen levels are dynamic during H_2_ production. In fact, the previous report showed evidence for both glycogen synthesis and degradation during the H_2_ production and demonstrated that the maximum theoretical reductant derived from glycogen oxidation alone could not account for the rate of H_2_ production measured^12^.

The current study did not directly investigate nitrogen fixation or diel-regulated cell cycling. Hence, the presented results are not assumed to be ubiquitous and there is no precedent to infer that these extend toward nitrogen fixation in natural ecosystems or N_2_-replete conditions. Also, it has been proposed that *Cyanothece* 51142 possesses temporal gene regulation processes which operate as circadian clocks^15^,^16^,^17^,^23^,^24^. This study does not refute that concept; in fact, the experiments here avoided external temporal cues by incubating *Cyanothece* 51142 cells exclusively under constant light through a period of weeks.

In summary, photolytic hydrogen production by autotrophic cyanobacteria represents a promising source of clean, renewable energy. This has been the focus of many fundamental and applied research investments made by both private enterprises and government funding agencies. However, significant developments still need to be made on the basic physiological understanding of these processes before bioprocess optimization may take full effect. This study enables an advance in the field though high resolution multi-omics analyses coupled with detailed kinetics which present a direct relationship between photosynthetic energy acquisition and nitrogenase enzyme activity. These insights bring us closer to resolving the mechanistic underpinnings of energy metabolism used to drive the complex nitrogenase machine for biotechnology that may ultimately enable robust and efficient biophotolytic conversion processes for the production of fuel from H_2_O, atmospheric CO_2_ and sunlight.

## Methods

### Media and culturing conditions

*Cyanothece* 51142 was cultured in modified ASP-2 medium^27^, supplemented with 0.75 mM NH_4_CL, 0.03 mM FeCl_3_ and 0.75 mM K_2_HPO_4_. Photobioreactor (PBR) cultures were operated by a previously described method^12^. Briefly, di-chromatic (680 and 630 nm LEDs) PBRs were operated as nitrogen limited chemostats with a 5.5 L working volume diluted at a 0.05 hr^−1^, 30 °C, pH 7.5 and controlled for constant incident and transmitted irradiance (250 and 10 μmol photons m^−2^ sec^−1^, respectively). Cells were never exposed to dark conditions during these experiments. The PBR cultures were sparged at 4.08 L min^−1^ with 1.3% CO_2_ in argon. H_2_ production profiles were initiated by arresting flow to the PBR while maintaining sparging with 1.3% CO_2_ in argon. Steady-state biomass concentrations were measured directly as ash-free cell dry weight (g_CDW_ L^−1^) as previously reported^25^.

### Transcriptomics

Global RNA sequencing was performed by a previously reported method utilizing SOLiD^TM^ sequencing technology^26^. RNA was extracted from *Cyanothece* 51142 cells collected at different time points from the bioreactor using TRIzol® Reagent (Invitrogen), followed by genomic DNA removal and cleaning using RNase-Free DNase kit and Mini RNeasy™ kit (Qiagen). The 50-base short read sequences produced by the 5500XL SOLiD^TM^ sequencer were mapped in color space using SOLiD^TM^ LifeScope^TM^ software version 2.5 using the default parameters against the genome of *Cyanothece* sp. ATCC 51142 (http://www.ncbi.nlm.nih.gov/genome). Resulting BAM files were then analyzed with the Rockhopper program as described previously^27^ to determine gene expression levels (RPKM).

### Proteomics

Global samples were measured via previously reported LC-MS methods^28^. Briefly, an accurate mass and time (AMT) tag approach was used that utilized tandem mass spectrometry to generate a reference peptide database of observed peptides. Samples were analyzed using an LTQ-Orbitrap Velos^TM^ (Thermo Fisher Scientific) MS interfaced with a reverse phase HPLC system for peptide separation (LC-MS). Only peptides unique in identifying a single protein were used; however, the uniqueness criteria was dropped for analysis of the subset of PS II (PsbA and PsbD) proteins to accommodate for the high degree of similarity between isoforms. Only proteins represented by >2 unique peptides were considered. All AMT values are represented as the mean from duplicate samples.

### Clustering and expression profiling

All filtering, clustering and profile analyses were performed with custom scripts (available upon request) written in Matlab and the Bioinformatics Tool Box (Mathworks). The steps used to filter expression profiles are detailed in a supplementary file. Hierarchical clustering was performed on the filtered mRNA data and grouped into six distinguished clusters. Matching protein and mRNA expression profiles synchronized by using K-means clustering to identify six distinct clusters (both protein and mRNA abundances) containing identical genes. The detailed algorithm for synchronized K-means clustering is described in the supplementary file. Eigen-gene and protein profiles are represented as the centroid of all profiles contained in a cluster.

### Statistics

Statistical significance (p < 0.05) in the proteomic data sets was determined by the Dunnett Test used to compare expression to the steady-state preconditions. Enrichment, within the transcriptomic and proteomic data sets, is defined as the percentage of genes within the profile for which a function has been assigned being significantly higher than the percentage of genes of the same function in the entire genome with a p-value of  <0.05 according to Fisher’s exact test.

## Additional Information

**How to cite this article**: Bernstein, H. C. *et al.* Multi-Omic Dynamics Associate Oxygenic Photosynthesis with Nitrogenase-Mediated H_2_ Production in *Cyanothece* sp. ATCC 51142. *Sci. Rep.* **5**, 16004; doi: 10.1038/srep16004 (2015).

## Supplementary Material

Supplementary Information

Supplementary Data
