# Supplementary material for: Multi-Omic Dynamics Associate Oxygenic Photosynthesis with Nitrogenase-Mediated H2 Production in Cyanothece sp. ATCC 51142
Source: Sci Rep. 2015 Nov 3;5:16004. doi: 10.1038/srep16004 (PMC4630603; doi:10.1038/srep16004)
Supplement: Supplementary Information [file srep16004-s1.pdf]

Supplementary file for:

Multi-Omic Dynamics Associate Oxygenic Photosynthesis with Nitrogenase-Mediated H_2 Production in *Cyanothece* sp. ATCC 51142

Authors: Hans C. Bernstein[1,2]*, Moiz A. Charania[2], Ryan S. McClure[2], Natalie C. Sadler[2], Matthew R. Melnicki[2], Eric A. Hill[2], Lye Meng Markillie[2], Carrie D. Nicora[2], Aaron T. Wright[2], Margaret F. Romine[2], Alexander S. Beliaev[2]*

Affiliations: [1]Chemical and Biological Signature Science, Pacific Northwest National Laboratory, Richland, WA 99352; [2]Biological Sciences Division, Pacific Northwest National Laboratory, Richland WA, 99352;

****Corresponding Authors:*** Hans C. Bernstein, Chemical and Biological Signature Science, Pacific Northwest National Laboratory, P. O. Box 999, MSIN: P7-50, Richland, WA 99352, phone: (509) 375-4518, email: hans.bernstein@pnnl.gov
Alexander S. Beliaev, Biological Sciences Division, Pacific Northwest National Laboratory, P. O. Box 999, MS: P7-50, Richland, WA 99352, phone: (509) 376-6966, email: alex.beliaev@pnnl.gov

1.1 Biomass and chlorophyll contents

Chlorophyll *a* (Chl) concentration of the cells was determined spectrophotometrically by a previously described method[1]. Chl *a* concentrations, in µg of Chl ml^{-1} were calculated by Equation S1.

$$[Chl] = 14.97(A_{678} - A_{730}) - 0.615(A_{625} - A_{730}) \qquad \text{Eq. S1}$$

As observed in a related study [2], Chl a concentrations remained essentially constant at 0.95 ± 0.11 mg L^{-1} from the steady-state through the end of the H_2 production.

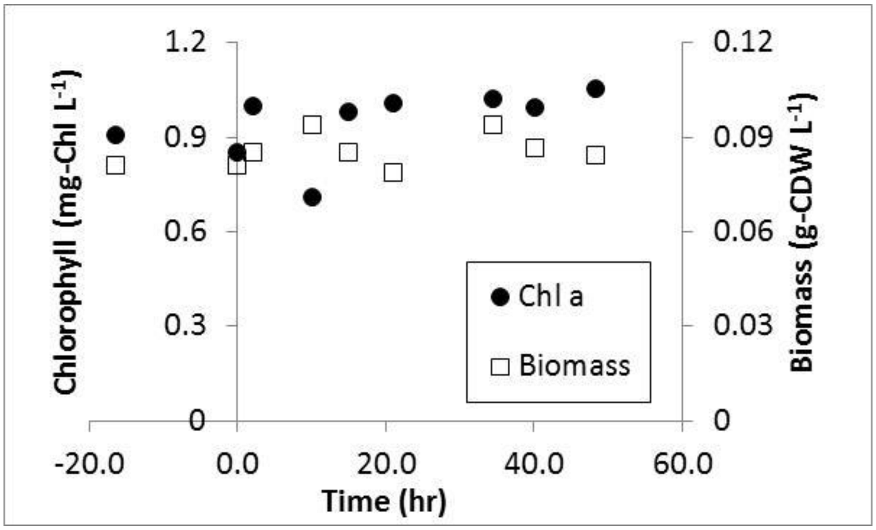

Figure S1. Chl *a* and ash-free dry weight biomass measurements over the H_2 production profile. Time zero indicates onset of nitrogen-depletion and absence of media addition (dilution rate = 0). Times prior to t = 0 represent measurements taken during the ammonia limited chemostat controlled steady-state.

1.2 Measurements of chlorophyll fluorescence. Variable Chl *a* fluorescence and fluorescence kinetic traces were measured by a previously reported methods[3]. Briefly, cells were sampled from the photobioreactor and dark-adapted for 5 min prior to taking measurements via pulse amplitude-modulated fluorometry (PAM) in a DUAL-PAM-100 equipped with a photodiode detector and RG665 filter (Walz GmbH, Effeltrich, Germany). Variable Chl *a* fluorescence parameters ($rETR_{max}$ and Fv/Fm) were measured and interpreted via standard approaches[4]. The slope of the post-illumination fluorescence rises (df/dt) are proxy measurements for the rate of cyclic electron transport (rCEF)[3,5] and were only observed (qualitatively) from the ammonium-limited chemostat, precondition (Fig. S2).

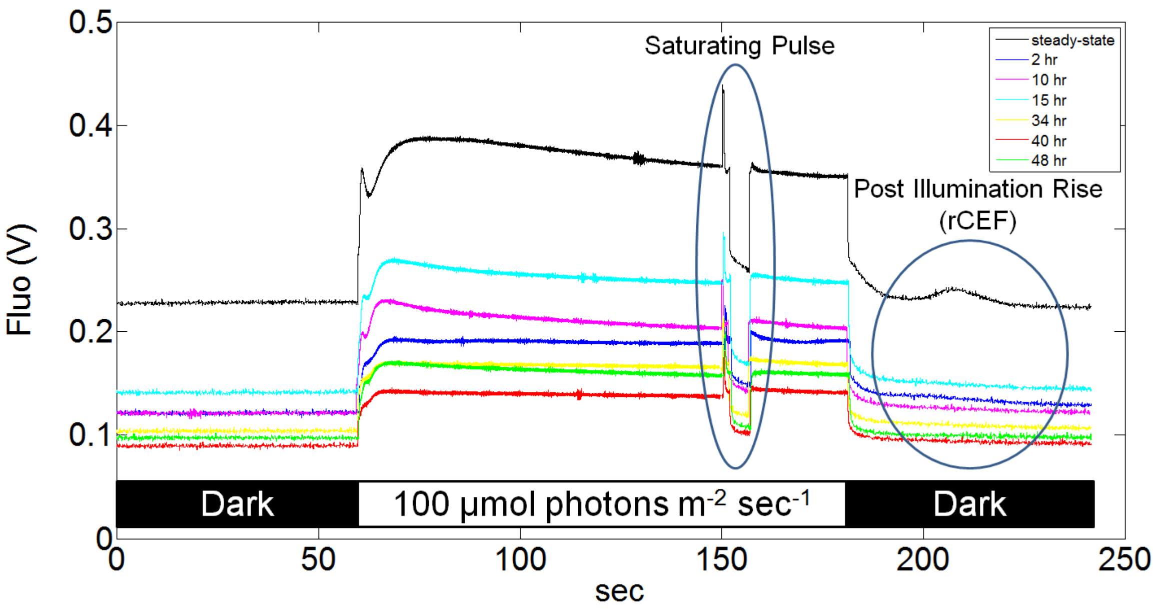

Figure S2. Chl *a* fluorescence traces measured from cells taken at different time points across the H_2 production transient. The post illumination rise in fluorescence is a result from reduction of plastoquinone from NAD(P)H or other reductants accumulated during illumination.

1.3 Mass balance of biological H_2 and O_2 production. The specific rates of net H_2 production and O_2 production were calculated from non-steady state mass balances represented in the following equation progression (Equations S3-S4). Simplified versions for the following equations were used for the steady-state reaction rates; q_{H2} and q_{O2}. Changes to the control volume, due to sampling, were considered negligible. The solute (H_2 or O_2) is represented by the symbol *x*; the k_la values were experimentally measured to be 55 and 74 hr^{-1} for O_2 and H_2 gas, respectively.

$$x\frac{dV}{dt} + V\frac{dx}{dt} = D \cdot V\left(x_{aq}^{in} - x_{aq}^{out}\right) + k_l^x a \cdot V\left(x_{gas}^{in} - x_{gas}^{out}\right) + R_{net}^x \cdot V \qquad \text{Eq. S2}$$

The following assumptions and simplifications were made based on the reaction geometry to yield Equation S4: (i) changes to the control volume, due to sampling, were considered negligible; (ii) and the dilution rate (*D*) was zero; (iii) the variable $x^{in}{}_{gas}$ (gas phase concentration) was zero; for both H_2 and O_2.

$$R_{net}^x = k_l^x a\left(x_{gas}^{out}\right) + \frac{dx}{dt} \qquad \text{Eq. S3}$$

A finite difference approximation was substituted into Equation S3 and both sides of the equation were normalized by the corresponding biomass (g-CDW) to yield the final working form of an equation for the specific rates production; q_{H2} or q_{O2} (Equation S4). The parameter *h*

is defined as the time difference between sample steps normalized by the total number of samples.

$$q_x(t) \approx k_l^x a\left[x_{gas}^{out}(t)\right] + \frac{x(t)-x(t-1)}{h} \qquad \text{Eq. S4}$$

2. Supplementary transcriptional and translational dynamics

2.1 Filtering of mRNA and protein expression data

All filtering and clustering was performed by custom Matlab (Mathworks) scripts that are available on request.

2.1.1 Standard mRNA expression filtering algorithm

Messenger RNA expression profiles were filtered with the custom Matlab script *mRNA_preproc_1* which implements the following steps (in order): 1) genes with profile expression variances in the bottom 10% were masked, 2) genes with absolute expression profiles within the bottom 20% (RPKM values) were masked, 3) RPKM values equal to zero were assigned an arbitrarily low value of 0.5, 4) expression profiles with the 20% highest level entropy [6] were masked, 5) each expression value was normalized to its corresponding steady-state condition and log2 transformed.

2.1.2 Standard protein expression filtering algorithm

Protein expression profiles were filtered with the custom Matlab script *mRNA_preproc_3* which implements the following steps (in order): 1) proteins with profile expression variances in the bottom 10% were masked, 2) protein expression profiles with the 20% highest level entropy [6] were masked, 5) each expression value was normalized to its corresponding steady-state condition and log2 transformed.

2.2 Synchronized profiles of protein and mRNA expression

These profiles were identified and calculated with the custom Matlab script *systiter_V2* which implements the following steps (in order): 1) protein expression profiles were filtered (see 2.1.2.) and organized by K-means into six groups, 2) each cluster was assigned a numeric cluster ID (1-6), 3) the mRNA expression files and gene IDs were collected from each protein cluster, 4) the mRNA expression profiles exhibiting variance across the profile in the bottom 10% were masked, and the corresponding gene IDs were logged, 4) protein expression profiles corresponding to genes that were masked in from the gene variance filter were also masked. This

algorithm output six clusters of protein and mRNA expression profiles containing identical coding genes.

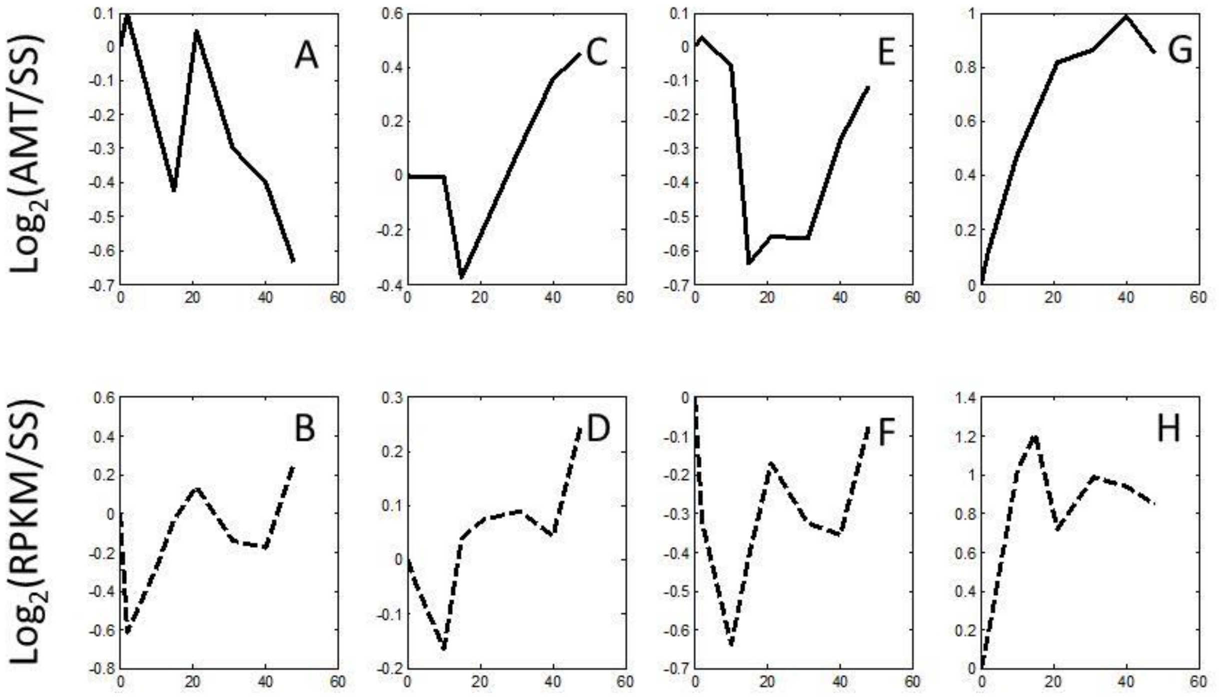

Figure. S3. K-means clustering of the relative protein abundances given in accurate mass and time (AMT) normalized by steady-state abundance. Plots represented as solid lines represent four clusters (mean value) calculated from 557 unfiltered protein profiles (of 1022). Dashed lines represent the mean value taken from the analogous gene transcripts. A and B) 159 proteins/transcripts enriched for TCA cycle second carbon oxidation processes ($p < 0.05$); C and D) 162 proteins/transcripts enriched for PS II main subunits (does not include PsbA or PsbD; $p < 0.05$); E and F) 88 proteins/transcripts enriched for PS I main subunits, Calvin-Benson cycle and Embden-Meyerhof processes ($p < 0.02$); G and H) 148 proteins/transcripts enriched for nitrogen fixation (including NifHDK; $p < 0.00001$). Abbreviation, SS, denotes the abundance measured at steady-state.

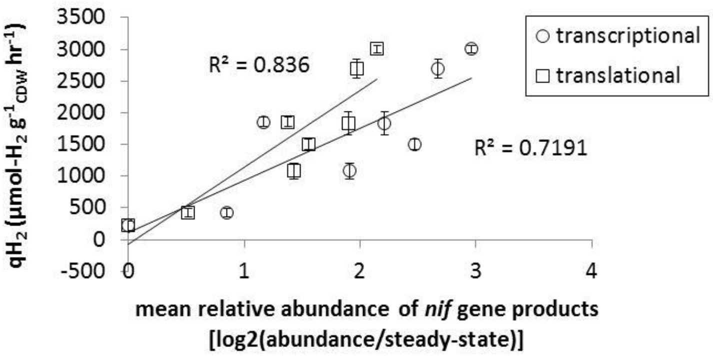

Figure S4. The specific rate of H_2 production plotted against the relative abundance profiles of *nif* gene transcripts (20 genes; open circles) and Nif proteins (12 proteins; open squares). Each relative abundance sample represents the mean taken at each time sample. Each specific rate value represents the mean over < 5 of continuously data logged measurements (minute sampling frequency). Error bars represent ± 1 standard deviation. R^2 values correspond to linear regression of each data set and are indications for proportionality.

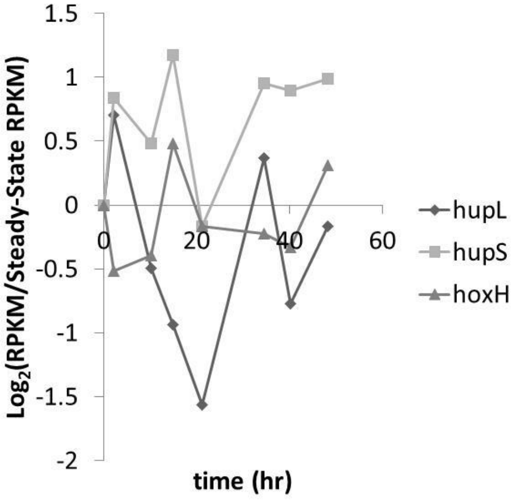

Figure S5. Relative abundance profiles of uptake (hup) and bi-directional (hox) hydrogenase transcripts.

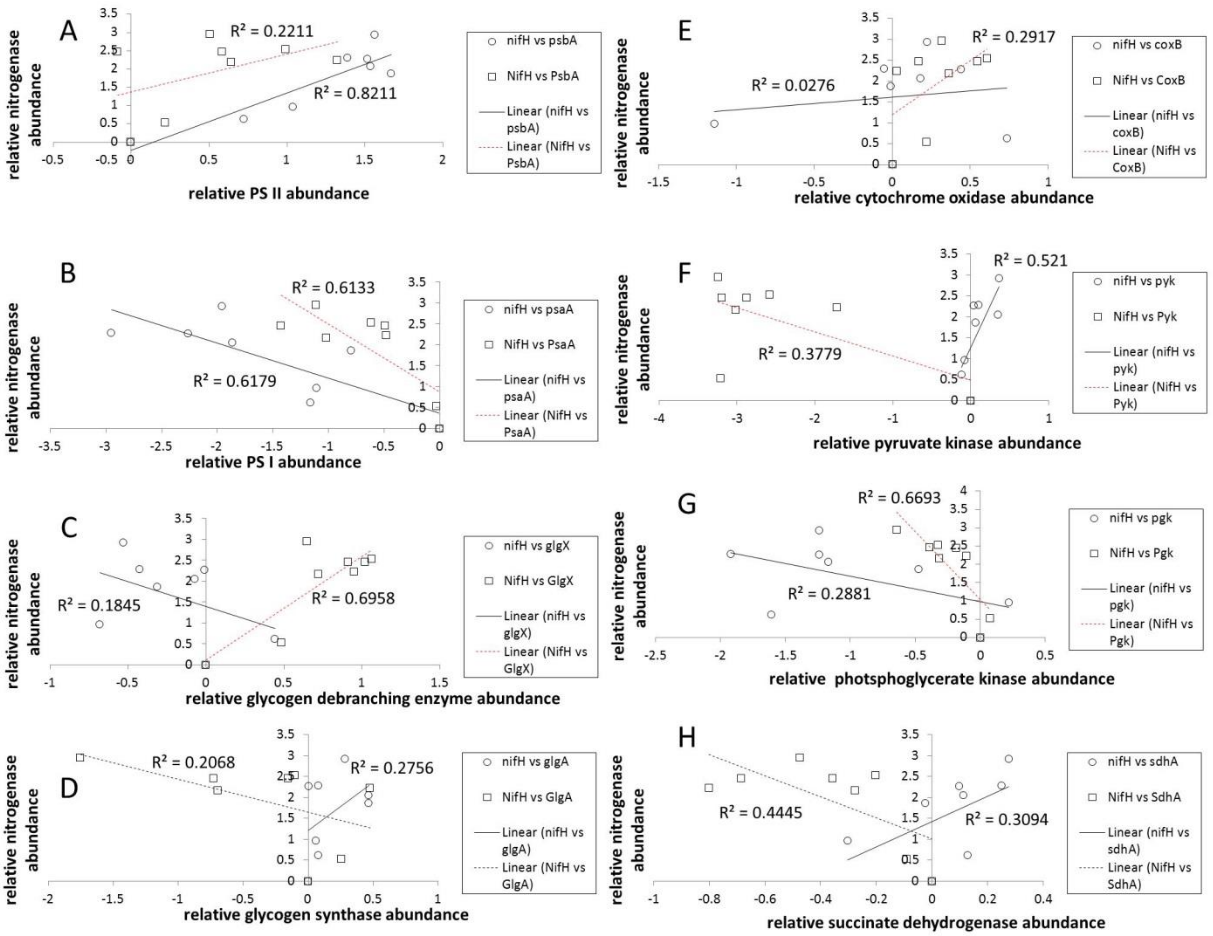

Figure S6. Linear correlations between the relative abundance profiles of NifH transcripts [$\log_2$(RPKM/steady-state RPKM); solid lines] and proteins [$\log_2$(AMT/steady-state AMT); dashed red lines]. The following correlations were considered: A) PsbA as a proxy for PS II, B) PsaA as a proxy for PS I, C) GlgX as a proxy for glycogen degradation, D) GlgA as a proxy for glycogen synthesis, E) CoxB as a proxy for respiration and oxidative phosphorylation, F) Pyk as a proxy for glycolysis and glycogen catabolism, G) Pgk as a second proxy for glycolysis and glycogen catabolism, and H) SdhA as a proxy for oxidative glycogen catabolism.

Table S1. Functional enrichment of mRNA clusters

RNA Modules with Argon and CO_2	Subsystem	p-value	Ratio	% Module	% Genome
1_red	peptidoglycan biosynthesis	0.0304	4.8840	1.6575	0.3394
1_red	phylloquinone biosynthesis via SEPHCHC	0.0409	7.3260	1.1050	0.1508
1_red	lipopolysaccharide export system LptABCDEFG	0.0149	14.6519	1.1050	0.0754
2_blue	photosystem II other common subunits	0.0057	3.7431	0.9174	0.2451
2_blue	plastoquinol/plastocyanin reductase (cytochrome b6-f complex)	0.0369	3.3792	0.5734	0.1697
2_blue	photosystem I main subunits	0.0034	6.0826	0.6881	0.1131
2_blue	phosphate signalling cascade PhoBRU-PstABCS	0.0410	4.0550	0.4587	0.1131
2_blue	lycopene biosynthesis from isopentyl pyrophosphate (IPP)	0.0410	4.0550	0.4587	0.1131
2_blue	glucosylglycerol biosynthesis	0.0403	6.0826	0.3440	0.0566
2_blue	phycocyanin biosynthesis	0.0055	5.2136	0.6881	0.1320
2_blue	photosystem II other subunits in cyanobacteria	0.0403	6.0826	0.3440	0.0566
2_blue	photosynthetic electron transport	0.0492	3.0413	0.5734	0.1885
3_yellow	pentose phosphate pathway_ oxidative phase	0.0152	9.1448	0.5172	0.0566
3_yellow	phycobilisome turnover	0.0366	5.4869	0.5172	0.0943

3_yellow	NAD(P)H:quinone oxidoreductase (complex I) NdhABCDEFGHIJKLMNOPQS	0.0105	3.0483	1.3793	0.4525
3_yellow	uridine monophosphate biosynthesis from glutamine	0.0119	4.5724	0.8621	0.1885
3_yellow	tRNA aminoacylation	0.0439	2.6128	1.0345	0.3959
3_yellow	Nucleotide excision repair	0.0246	6.8586	0.5172	0.0754
4_magenta	polymorphic toxins	0.0386	1.9579	2.3256	1.1878
4_magenta	polysaccharide biosynthesis	0.0447	2.5698	1.1628	0.4525
4_magenta	sulfur modification of DNA	0.0388	5.1395	0.5814	0.1131
5_green	uridine monophosphate biosynthesis from glutamine	0.0395	7.2658	1.3699	0.1885
5_green	lipoic acid metabolism	0.0100	18.1644	1.3699	0.0754
5_green	protease_ periplasmic	0.0068	24.2192	1.3699	0.0566
5_green	NAD(P) biosynthesis common pathway from nicotinic acid mononucleotide route 1	0.0041	36.3288	1.3699	0.0377
6_black	ClpP1-ClpP2-ClpX proteolytic complex	0.0136	5.1797	0.4883	0.0943
6_black	ribosome small subunit	0.0039	2.9598	1.1719	0.3959
6_black	RNA polymerase RpoABCEZ	0.0435	4.1438	0.3906	0.0943
6_black	photosystem II main subunits	0.0442	2.8253	0.5859	0.2074
6_black	Nitrogen fixation	0.0000	4.7293	2.0508	0.4336

Table S2. Functional enrichment of protein clusters

Protein (k-means) clusters	Subsystem	p-value	Ratio	% Module	% Genome
1	pentose phosphate pathway_ oxidative phase	0.0026	39.7303	2.2472	0.0566
1	translation elongation factor	0.0327	59.5955	1.1236	0.0189
1	Double strand breaks repair	0.0134	13.2434	2.2472	0.1697
1	ribosome large subunit	0.0000	14.8989	8.9888	0.6033
1	citrate cycle_ 2-OGDH bypass	0.0487	29.7978	1.1236	0.0377
1	biosynthesis of UDP-N-acetyl-D-mannosamine (UDP-mannAc)	0.0327	59.5955	1.1236	0.0189
1	allophycocyanin biosynthesis	0.0071	19.8652	2.2472	0.1131
1	chaperone system: GroEL/GroES	0.0026	39.7303	2.2472	0.0566
1	TCA cycle second carbon oxidation	0.0487	29.7978	1.1236	0.0377
1	phycocyanin biosynthesis	0.0090	17.0273	2.2472	0.1320
1	cell division	0.0487	29.7978	1.1236	0.0377
1	acetyl-CoA biosynthesis from acetate_ direct	0.0327	59.5955	1.1236	0.0189
1	rRNA transcription factors	0.0001	59.5955	3.3708	0.0566
1	photosynthetic electron transport	0.0011	17.8787	3.3708	0.1885
1	Nitrogen fixation	0.0086	7.7733	3.3708	0.4336

2	histidine biosynthesis from ribose-5-phosphate	0.0314	8.5000	1.2821	0.1508
2	photosystem I high light stabilizing complex	0.0113	17.0000	1.2821	0.0754
2	Calvin-Benson cycle	0.0024	14.5714	1.9231	0.1320
2	NAD(P)H:quinone oxidoreductase (complex I) NdhABCDEFGHIJKLMNOPQS	0.0002	8.5000	3.8462	0.4525
2	purine (inosine-5'-phosphate) biosynthesis from ribose-5-phosphate	0.0081	8.5000	1.9231	0.2262
2	ClpP1-ClpP2-ClpX proteolytic complex	0.0155	13.6000	1.2821	0.0943
2	phosphate homeostasis	0.0203	11.3333	1.2821	0.1131
2	Embden-Meyerhof pathway	0.0024	14.5714	1.9231	0.1320
2	photosystem II biogenesis	0.0004	15.1111	2.5641	0.1697
2	photosystem II main subunits	0.0066	9.2727	1.9231	0.2074
2	threonine biosynthesis from homoserine	0.0077	22.6667	1.2821	0.0566
2	DNA gyrase modulation	0.0077	22.6667	1.2821	0.0566
3	uridine monophosphate biosynthesis from glutamine	0.0495	6.3904	1.2048	0.1885
3	pyruvate dehydrogenase complex PdhABCD-AceE	0.0127	15.9759	1.2048	0.0754
3	tRNA aminoacylation	0.0010	7.6076	3.0120	0.3959
3	F1/F0 ATPase AtpABCDEFGH	0.0063	9.5855	1.8072	0.1885
3	branched chain amino acid biosynthesis	0.0014	19.1711	1.8072	0.0943

3	Plastoquinol plastocyanin reductase (cytochrome b6-f complex)	0.0049	10.6506	1.8072	0.1697
3	ClpP1-ClpP2-ClpX proteolytic complex	0.0014	19.1711	1.8072	0.0943
3	chlorophyllide a biosynthesis from protoporphyrin IX	0.0421	7.1004	1.2048	0.1697
3	ribosome large subunit	0.0226	3.9940	2.4096	0.6033
3	photosystem I main subunits	0.0001	21.3012	2.4096	0.1131
3	ornithine biosynthesis from glutamate_ acetylated branch	0.0127	15.9759	1.2048	0.0754
3	Calvin-Benson cycle_ Embden-Meyerhof pathway	0.0127	15.9759	1.2048	0.0754
3	ribosome small subunit	0.0063	6.0861	2.4096	0.3959
3	RNA polymerase RpoABCEZ	0.0001	25.5614	2.4096	0.0943
3	4_6-dideoxy-4-oxo-dTDP-D-glucose biosynthesis from glucose-1-phosphate	0.0086	21.3012	1.2048	0.0566
3	glycogen metabolism	0.0174	12.7807	1.2048	0.0943
3	citrate cycle_ first carbon oxidation	0.0086	21.3012	1.2048	0.0566
3	IPP biosynthesis via deoxyxylulose	0.0286	9.1291	1.2048	0.1320
4	chorismate biosynthesis via shikimate	0.0135	14.6317	1.3793	0.0943

4	ribosome large subunit	0.0004	6.8586	4.1379	0.6033
4	peroxide detoxification	0.0330	8.1287	1.3793	0.1697
4	ribosome small subunit	0.0001	10.4512	4.1379	0.3959
4	translation elongation	0.0135	14.6317	1.3793	0.0943
4	chaperone system: DnaK-DnaJ-GrpE	0.0135	14.6317	1.3793	0.0943
4	phycobilin biosynthesis	0.0067	24.3862	1.3793	0.0566
4	photosynthetic electron transport	0.0390	7.3159	1.3793	0.1885
4	Nitrogen fixation	0.0000	15.9040	6.8966	0.4336

References Cited

1 Min, H. & Sherman, L. A. Hydrogen production by the unicellular, diazotrophic cyanobacterium Cyanothece sp. strain ATCC 51142 under conditions of continuous light. *Applied and environmental microbiology* **76**, 4293-4301 (2010).

2 Melnicki, M. R. *et al.* Sustained H2 production driven by photosynthetic water splitting in a unicellular cyanobacterium. *MBio* **3**, e00197-00112 (2012).

3 Bernstein, H. C. *et al.* Effect of mono-and dichromatic light quality on growth rates and photosynthetic performance of Synechococcus sp. PCC 7002. *Frontiers in microbiology* **5** (2014).

4 Schreiber, U. in *Chlorophyll a Fluorescence* 279-319 (Springer, 2004).

5 Deng, Y., Ye, J. & Mi, H. Effects of low CO2 on NAD (P) H dehydrogenase, a mediator of cyclic electron transport around photosystem I in the cyanobacterium Synechocystis PCC6803. *Plant and cell physiology* **44**, 534-540 (2003).

6 Liu, H., Li, J. & Wong, L. A comparative study on feature selection and classification methods using gene expression profiles and proteomic patterns. *Genome informatics* **13**, 51-60 (2002).
